# Combining single-cell transcriptomics and CellTagging to identify differentiation trajectories of human adipose-derived mesenchymal stem cells

**DOI:** 10.1186/s13287-023-03237-3

**Published:** 2023-02-01

**Authors:** Kai Lin, Yanlei Yang, Yinghao Cao, Junbo Liang, Jun Qian, Xiaoyue Wang, Qin Han

**Affiliations:** 1 State Key Laboratory of Medical Molecular Biology, Department of Biochemistry and Molecular Biology, School of Basic Medicine Peking Union Medical College, Institute of Basic Medical Sciences Chinese Academy of Medical Sciences, Beijing, China; 2 Beijing Key Laboratory of New Drug Development and Clinical Trial of Stem Cell Therapy (BZ0381), School of Basic Medicine Peking Union Medical College, Peking Union Medical College Hospital, Institute of Basic Medical Sciences Chinese Academy of Medical Sciences, Beijing, China

**Keywords:** MSCs, Heterogeneity, Single-cell RNA-seq, CellTagging, Osteogenic differentiation

## Abstract

**Background:**

Mesenchymal stromal cells (MSCs) have attracted great attention in the application of cell-based therapy because of their pluripotent differentiation and immunomodulatory ability. Due to the limited number of MSCs isolated from donor tissues, a large number of MSCs need to be expanded in a traditional two-dimensional cell culture device to obtain a sufficient therapeutic amount. However, long-term cultivation of MSCs in vitro has been proven to reduce their differentiation potential and change their immunomodulatory characteristics. We aimed to explore the cellular heterogeneity and differentiation potential of different MSCs expanded in vitro and reconstruct the complex cloning track of cells in the process of differentiation.

**Methods:**

Single cell transcriptome sequencing was combined with ‘CellTagging’, which is a composite barcode indexing method that can capture the cloning history and cell identity in parallel to track the differentiation process of the same cell over time.

**Results:**

Through the single-cell transcriptome and CellTagging, we found that the heterogeneity of human adipose tissue derived stem cells (hADSCs) in the early stage of culture was very limited. With the passage, the cells spontaneously differentiated during the process of division and proliferation, and the heterogeneity of the cells increased. By tracing the differentiation track of cells, we found most cells have the potential for multidirectional differentiation, while a few cells have the potential for unidirectional differentiation. One subpopulation of hADSCs with the specific osteoblast differentiation potential was traced from the early stage to the late stage, which indicates that the differentiation trajectories of the cells are determined in the early stages of lineage transformation. Further, considering that all genes related to osteogenic differentiation have not yet been determined, we identified that there are some genes that are highly expressed specifically in the hADSC subsets that can successfully differentiate into osteoblasts, such as Serpin Family E Member 2 (*SERPINE2*), Secreted Frizzled Related Protein 1 (*SFRP1*), Keratin 7 (*KRT7*), Peptidase Inhibitor 16 (*PI16)*, and Carboxypeptidase E (*CPE*), which may be key regulatory genes for osteogenic induction, and finally proved that the *SERPINE2* gene can promote the osteogenic process.

**Conclusion:**

The results of this study contribute toward the exploration of the heterogeneity of hADSCs and improving our understanding of the influence of heterogeneity on the differentiation potential of cells. Through this study, we found that the *SERPINE2* gene plays a decisive role in the osteogenic differentiation of hADSCs, which lays a foundation for establishing a more novel and complete induction system.

**Supplementary Information:**

The online version contains supplementary material available at 10.1186/s13287-023-03237-3.

## Introduction

Mesenchymal stromal cells (MSCs) are multipotent cells with the ability of self-renewal and differentiation [1]. Studies have shown that MSCs can differentiate into various cell lineages, such as osteoblasts, adipocytes, and chondrocytes [2–4]. Compared with embryonic stem cells and induced pluripotent stem cells, MSCs have lower risk of immunogenic rejection and teratoma formation, and its use does not involve ethical problems [5]. Therefore, stem cell therapy with MSCs may be a potential treatment method for intractable and uncontrollable diseases and has shown some promising results in preclinical studies. A variety of alternative tissue sources of MSCs have been identified, including bone marrow, adipose tissue, umbilical cord, Wharton’s jelly, and solid organs [6]. Most studies make use of bone marrow-derived mesenchymal stem cells (BMSCs) because of their potential to differentiate into various mesenchymal tissues and their immunomodulatory functions. However, as the source of MSCs, bone marrow is limited by the invasive and painful suction process, and the abundance of BMSCs is usually 0.001–0.01% of cells [3]. Among other sources, adipose tissue has been identified as an ideal source for isolating MSCs, and the average frequency of obtaining MSCs via treated liposuction is 2% [7] because, compared with other sources, it can be easily obtained in large quantities through minimally invasive surgery [8]. After separation, these so-called adipose-derived stromal cells (ADSCs) can be expanded in vitro and have the potential for multi-lineage differentiation in vitro [9].

The International Society of Cell Therapy has put forward the most basic standard to clearly define human MSCs. The first criterion to be met for cells to be defined as MSCs is that they should possess the ability to adhere to a plastic surface under standard culture conditions. Secondly, a minimum percentage of cells must have specific surface marking characteristics, that is, more than 95% of the cells must express *CD105*, *CD73*, and *CD90*, but less than 2% of cells may express *CD45*, *CD34*, *CD14* or *CD11b*, *CD79* or *CD19,* and *HLA‐DR* [10]. Finally, cells must be able to differentiate into at least three different lineages under inductions in vitro, such as osteoblasts, adipocytes, and chondrocytes.

The heterogeneity of cells is a common feature of organisms. Cellular heterogeneity is not only influenced by the external microenvironment, but also have certain differences within the population [11]. Traditional two-dimensional (2D) cell culture has been the main method for medical and biological research and drug development for decades because of its simplicity and robustness [12]. Since there are limited number of MSCs in adult tissue, after being separated from donor tissue, the collected primary MSCs should be first expanded in a 2D culture device to achieve sufficient numbers for various in vitro modeling and therapeutic applications. However, long-term culture of MSCs in in vitro 2D systems has been shown to reduce their differentiation potential [13] and changes their phenotypes [14] and immunomodulatory properties [15], indicating that MSCs become a heterogeneous cell population of different lineages with passage. In order to explore the heterogeneity of MSCs and identify the differences in cell differentiation ability, it is necessary to study the gene expression of cells at the single cell level. In recent years, single cell transcriptome sequencing (scRNA-seq) technology has become a powerful tool for studying the heterogeneity of tissues and cells. It can compare the differences between cells, reveal the unique transcriptome characteristics of a single cell [16], and, thus, identify differential gene expression profiles in heterogeneous cell groups, providing unprecedented information without any prior knowledge of cell groups [17]. By separating single cells, capturing their transcripts, and generating a transcript sequencing library for each cell, scRNA-seq can evaluate the basic biological characteristics of cell populations with unprecedented resolution and reveal the unique subtle changes of each cell [18, 19].

However, scRNA-seq can only reveal the cell state at a single time point and cannot explain the process of cell state transition in detail. In order to analyze the transformation of the identity of the single cell and cell cloning level at the same time, we adopted a high-throughput cell tracking method, ‘CellTagging’. This technology is based on a lentivirus that can uniquely label a single cell using a combination of heritable CellTags. CellTags are highly expressed in cells and are easily captured within each single cell transcriptome so that the cloning history can be recorded with the passage time, parallel to the cell identity. By uniquely marking single cells with heritable barcode combinations, ‘CellTagging’ can simultaneously analyze the cell identity and cloning history of a cell [20, 21].

In this study, scRNA-seq was used to analyze the gene expression profiles of human adipose-derived MSCs cultured in vitro at different times and compare the heterogeneity changes of the cells during the process of in vitro expansion. After that, we induced differentiation of the cells cultured in vitro, and in combination with "CellTagging" technology, we tracked the early differentiation trajectory of hADSCs by labeling different cells with heritable barcode combinations. Through tracing, we demonstrated the cloning track of hADSC differentiation and made the successfully differentiated cells correspond to the original hADSCs. By comparing with the hADSCs that successfully or unsuccessfully differentiated, we could identify the cells that were more likely to successfully differentiate. Through comparisons, it is possible to understand the influence of cell heterogeneity on the differentiation track and guide the establishment and optimization of the differentiation system by intervening the gene expression of cells.

## Methods

### Isolation, culture, and differentiation of hADSCs

hADSCs were isolated from human adipose tissues obtained from liposuction donors. In our experiment, hADSCs at passage 3 were used. All experiments and procedures were approved by the Institutional Review Board of Institute of Basic Medical Sciences, Chinese Academy of Medical Sciences.

To differentiate into endoderm, hADSCs were seeded in a 6 cm^2^ cell culture dish with basic medium (BM) DMEM-F12 (Gibco, Grand Island, NY) containing 10% FBS (Gibco, Grand Island, NY) and EGF (20 ng/mL). On the second day, the medium was replaced with BM DMEM-F12 supplemented with 50 ng/mL Wnt3a (Peprotech, USA). Wnt3a was then withdrawn from the medium for the following 4 days. Then, 5 ng/mL Activin A (Peprotech, USA), EGF (20 ng/mL), and bFGF (10 ng/mL) were added to the whole differentiation system during the induction process.

For osteogenic differentiation, hADSCs were inoculated on the 6-well culture plate. When the cells fused and grew to 60–70%, the BM was changed to osteogenic medium (OM). The OM was DMEM-F12 (Gibco) supplemented with 2% FBS (Gibco), penicillin–streptomycin (100 ×), 10 nM dextramisone, 50 μM ascorbic acid, and 10 mM *β*-glycerophosphate. The OM was changed every 3 days, and osteogenic markers were detected on the 7th and 14th day after osteogenic induction.

### RNA extraction and qRT-PCR analysis

Total RNA was extracted from cells using the TRIzol Regent (Invitrogen, USA), and 2 μg of RNA was reverse-transcribed with oligo (dT) primer and M-MuLV Reverse Transcriptase (Takara, Japan). qRT-PCR was conducted on a QuantStudio™ Design & Analysis system (ABI, USA) using SYBR-Green Mastermix (YEASEN, China). The relative RNA levels were normalized to GAPDH using the 2^−ΔΔCt^ method. The primer sequences are listed in Additional file 2: Table S1.

### Western blot

Protein was extracted using RIPA buffer with Cocktail (1:100, Beyotime, China) and quantified using the BCA Protein Assay kit (Beyotime, China). Proteins in lysates were separated by NuPAGE 4–12% Bis–Tris Gel (invitrogen, USA) and transferred to polyvinylidene difluoride membranes (0.22 μm, Millipore, Danvers, MA, USA). The membranes were blocked with 5% milk for 1 h at room temperature, incubated with the primary antibody (Abcam, ab154591, SERPINE2; CST, 3700, *β*-Actin) overnight at 4 °C, and then incubated with the secondary antibodies (ZSGB-BIO, ZB-5305, Anti-mouse HRP-linked Antibody) at room temperature for 1 h. The proteins were detected using an ECL reagent (Millipore, USA).

### Lentivirus production

293 T cells were transferred together with the pSMAL-CellTag construct and packaging constructs pCMV-dR8.2 dvpr (Addgene plasmid 8455) and pCMV-VSVG (Addgene plasmid 8454) to produce lentiviral particles. The virus was harvested 24 and 48 h after transfection and filtered through a low-protein binding 0.45 μM filter before applying to cells.

### CellTagging methodology

To generate CellTagV1 libraries, we transfected the CellTagV1 plasmid into Stellar Competent Cells (Takara Biosciences, 636,764). The cells recovered after transformation were grown overnight in liquid culture, followed by maxi-prep extraction of the plasmid DNA. Extracted CellTagV1 plasmid was used to package the lentivirus genome, and the lentivirus was then to transduce hADSCs at a multiplicity of infection of 3–4. For 10 × genomics-based experiments, the initial hADSC population was transduced with CellTagsV1 for 24 h, then washed and further culture for 48 h. Afterward, cells were split with one portion taken for 10 × genomics-based experiments and two portions for induction. Following 5 days of induction, the cells were subjected to 10 × genomics-based experiments again. CellTagV1 libraries were deposited at Addgene: pSMAL-CellTag-V1 (https://www.addgene.org/115643) [22].

### 10×  Genomics procedure

For the preparation of single-cell library on the 10 × Genomics platform, according to the manufacturer’s instructions in the users’ guide of Chromium Single Cell 3′ Reagents Kits V2, the Chromium Single Cell 3′ Library & Gel Bead Kit v2 (PN-120237), Chromium Single Cell 3′ Chip kit v2 (PN-120236), and Chromium i7 Multiplex Kit (PN-120262) were used. Just before cell capture, the digested cells were placed on ice, then centrifuged at 3000 rpm for 5 min at 4 °C, and subsequently rehydrated in PBS, for capturing 10,000 single-cell transcriptomes. The obtained cDNA library was quantified on an Agilent Tapestation, and sequenced on an Illumina HiSeq 3000 [23].

### 10× Genomics data QC analysis

The Cell Ranger v2.1.0 pipeline was used to process data generated by 10 × Chromium platform. The pipeline was used with the reference genome. After this step, the default Cell Ranger pipeline was realized, and the filtered output data was used for downstream analyses. First, a small number (< 200) of cells with unique detection genes was removed. Then, cells whose total number of unique molecules (UMIs, after log10 transformation) was not within the range of three standard deviations of the average value were removed. Subsequently, by fitting a loess curve (span = 0.5, degree = 2) to the number of UMIs with the reading number as the predictor (after log10 transformation), the peripheral cells with abnormally high or low UMI numbers and the remaining cells with more than three standard deviations from the average were removed. This process was also used to remove cells with an abnormally high or low gene number. Finally, we removed cells with mitochondrial genes accounting for more than 10% of the UMI count.

### CellTag identification and clone calling

For the CellTag analysis method, we refer to two previously published articles [20, 22]. Since our induction system has a relatively short time, we only used the CellTagV1 library "CCGGTNNNNNNNNGAATTC" for lineage reconstruction and identification. The specific process is to extract cells’ barcodes and CellTag UMI matrix from the BAM files of 10 × genomics pipeline. The Read ID, Sequence, Cell Barcode, UMI, CellTag Sequence, and Aligned Genes were captured for each read, and filtered for downstream clone calling and lineage reconstruction analysis.

Cells with CellTags that did not appear on the whitelists generated for the CellTag plasmid library were removed from the CellTag matrix. Cells expressing more than 20 CellTags (which may correspond to multiple CellTags) and the cells expressing less than 2 CellTags were excluded. To identify clonally-related cells, the similarity of CellTag signatures between cells were calculated through Jaccard analysis using the R package Proxy. A Jaccard score of > 0.7 was used as a cutoff to identify cells highly likely to be related.

### Seurat analysis

After filtering and normalization, the R package Seurat⁠ was used for the clustering and visualization of cells. As the data were previously normalized, they were loaded into Seurat without normalization, scaling, or centering. In addition to expressing data, metadata for each cell, including information such as clone identity, was also collected. Seurat was used to remove unnecessary variation, and the number of UMIs and proportion of mitochondrial UMIs were regressed. Next, highly variable genes were identified by PCA and used as the input of dimensionality reduction. The PCs and related genes obtained were examined to determine the number of components in downstream analysis. Then, these PCs were used as input for clustering cells, and t-distributed stochastic neighbor embedding (t-SNE) was used to visualize these clusters.

### Availability of code

Code for processing CellTag data and clone-calling is available on GitHub (https://github.com/morris-lab).

### ALP staining

The phenotype of osteoblasts was assessed by measuring the activity of alkaline phosphatase (ALP). The differentiated cells were inoculated into 6-well plates at an initial density of 1 × 10^6^ cells/well, and were osteogenically differentiated in the osteogenic medium for 14 days. The cells were washed with PBS twice and fixed with 4% paraformaldehyde for 30 min at room temperature. ALP staining kit (BOSTER, AR1023) was used for stained cells at room temperature for 30 min. The number of mineralized nodules was calculated under a microscope.

### Alizarin red S staining

Alizarin Red staining was used to detect calcification in the late induction period. Differentiated cells were washed and fixed in 10% (v/v) formaldehyde (Solarbio, P1110) for 30 min. The fixed cells were stained with 2% alizarin red S solution (Sigma-Aldrich, A5533). After dyeing for 30 min at room temperature, cells were washed with distilled water. After staining, cells were observed with optical microscope and photographed.

### Data availability statement

The raw sequence data reported in this paper have been deposited in the Genome Sequence Archive (Genomics, Proteomics & Bioinformatics 2021) in National Genomics Data Center (Nucleic Acids Res 2022), China National Center for Bioinformation / Beijing Institute of Genomics, Chinese Academy of Sciences (GSA-Human: HRA003716) that are publicly accessible at https://ngdc.cncb.ac.cn/gsa-human.

### Statistical analysis

Each experiment was conducted at least three times. GraphPad Prism7 (GraphPad Prism, San Diego, Calif.) software was used for all statistical analysis, and it is expressed as the mean ± standard deviation. The statistical comparison between the two groups was made by Student’s *t*-test. Differences was considered to be statistically significant when *P < 0.05, **P < 0.01, and ***P < 0.001.

## Results

### Identification of hADSCs

**Fig. 1:**
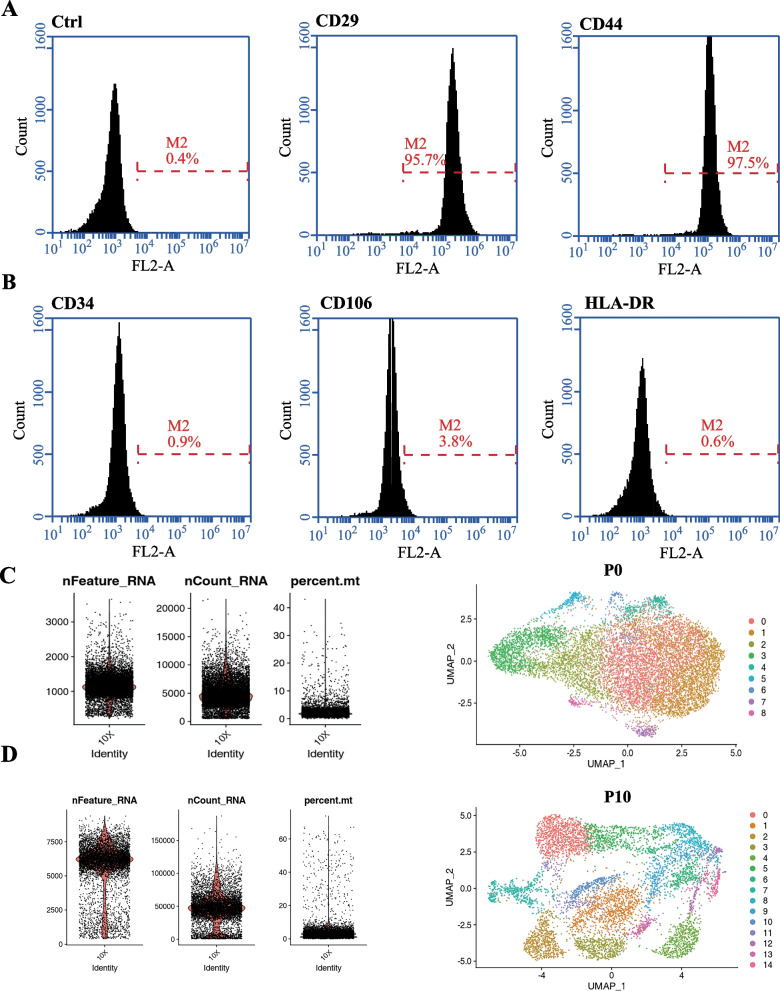
(**A**, **B**) Flow cytometric analysis of hADSCs derived from human adipose tissue. hADSCs positively express CD29 and CD44 but negatively express CD34, CD106, and HLA-DR. (**C**, **D**) Quality control of cells and UMAP visualizing of the results of P0 and P10 clustering, respectively

In order to identify hADSCs, we detected the cell surface markers. Classical hADSC markers include *CD44* and *CD29* were detected (Fig. 1A), and these cells lack the expression of *CD34*, *CD106*, and *HLA-DR* (Fig. 1B) [24]. We found that the expression of these markers was uniform and consistent. The results of flow cytometry analysis demonstrated that nearly 100% of gated cells expressed positive markers or lacked negative markers. The expression of all positive markers was always above 90%, and the expression of negative markers in the gated cells of different donors was below 5%.

### Cell population landscapes for the naive hADSCs (P0) and expanded hADSCs (P10)

We collected cells from immature hADSC population and the 10^th^ generation population (P0 and P10, respectively) after in vitro expansion. Large-scale single-cell RNA-seq (10 × Genomics) was performed on P0 and P10 after amplification. Cells with a clear outlying number of genes were perceived as potential multiples and excluded from subsequent analyses. Cells with 2000 + genes in the P0 sample and 10,000 + genes in the P10 sample were, therefore, excluded from downstream analysis. Similarly, cells with 10% or more mitochondrial gene proportions were excluded from the two samples. Totals of 10,112 and 6,844 cells were captured from the P0 (Fig. 1C) and P10 (Fig. 1D) samples, respectively. Statistically significant principal components were determined, and the two UMAP dimensions were approximated and projected by the Uniform Manifold Approximation and Projection (UMAP), resulting in nine clusters of P0 cells and 15 clusters of P10 cells. After cell expansion in vitro, we found that the distribution of the subgroups was relatively dispersed, and the gene expression of the different subgroups was quite different. This shows that, in the process of in vitro culture, the cells spontaneously differentiated with division from the original uniform state to a heterogeneous state, and the amplified hADSCs showed the characteristics of cell heterogeneity.

### Identifying the characteristics of candidate subgroups before and after hADSC expansion

**Fig. 2:**
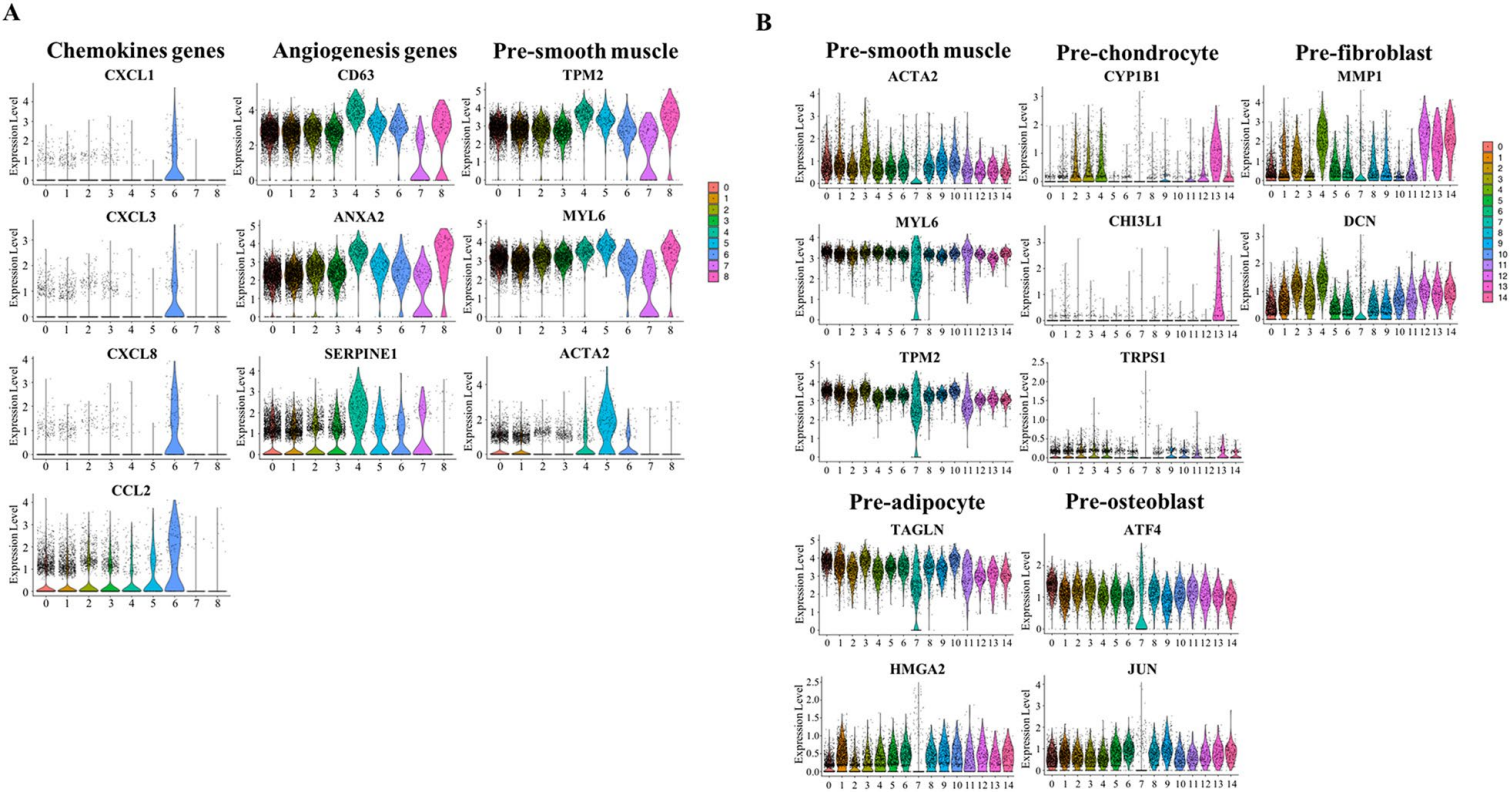
Violin plots showing differential gene expression and biological functions of several representative subpopulations of P0 (**A**) and P10 (**B**)

In order to identify the biological function and cell identity of each cluster, differential gene analysis was conducted to reveal the statistically significant gene ontology of each cluster. By performing these analyses, cell identities for all clusters were determined. In the P0 sample, we observed that the expression of the chemokine gene in cluster CC6 was more significant. Genes related to angiogenesis were highly expressed in cluster CC4, which indicated that this subgroup is related to angiogenesis. In addition, we found that cluster CC5 was related to the formation of smooth muscle. This subgroup highly expressed genes related to smooth muscle formation (Fig. 2A). In the P10 sample, cluster CC0 and CC3 expressed smooth muscle-related genes, such as Actin Alpha 2 (*ACTA2*) [25], Myosin Light Chain 6 (*MYL6*) [26], and Tropomyosin 2 (*TPM2*) [27]; cluster CC13 was enriched in Cytochrome P450 Family 1 Subfamily B Member 1 (*CYP1B1*) [28], Chitinase 3 Like 1 (*CHI3L1*) [29], and Transcriptional Repressor GATA Binding 1 (*TRPS1*), which were highly expressed in early chondrocytes; cluster CC4 showed high expression of fibroblast-related genes, such as Matrix Metallopeptidase 1 (*MMP1*) [30] and Decorin (*DCN*) [31], and adipocyte markers, such as Transgelin (*TAGLN*) [32] and High Mobility Group AT-Hook 2 (*HMGA2*) [33], were only expressed in cluster CC1. Clusters CC1, CC2, and CC5 were identified as the osteogenic differentiation clusters expressing the known genes Activating Transcription Factor 4 (*ATF4*) and Jun Proto-Oncogene, AP-1 Transcription Factor Subunit (*JUN*) [34] that regulate osteogenesis (Fig. 2B).

Compared with the P0 sample, many new functional subgroups appeared after the expansion of the cells [35]. This remarkable result indicates that, in the process of proliferation in vitro, the cells spontaneously differentiate and the identity characteristics of the cells change in many aspects.

### Cloning-related cell distribution of the P10 sample

**Fig. 3:**
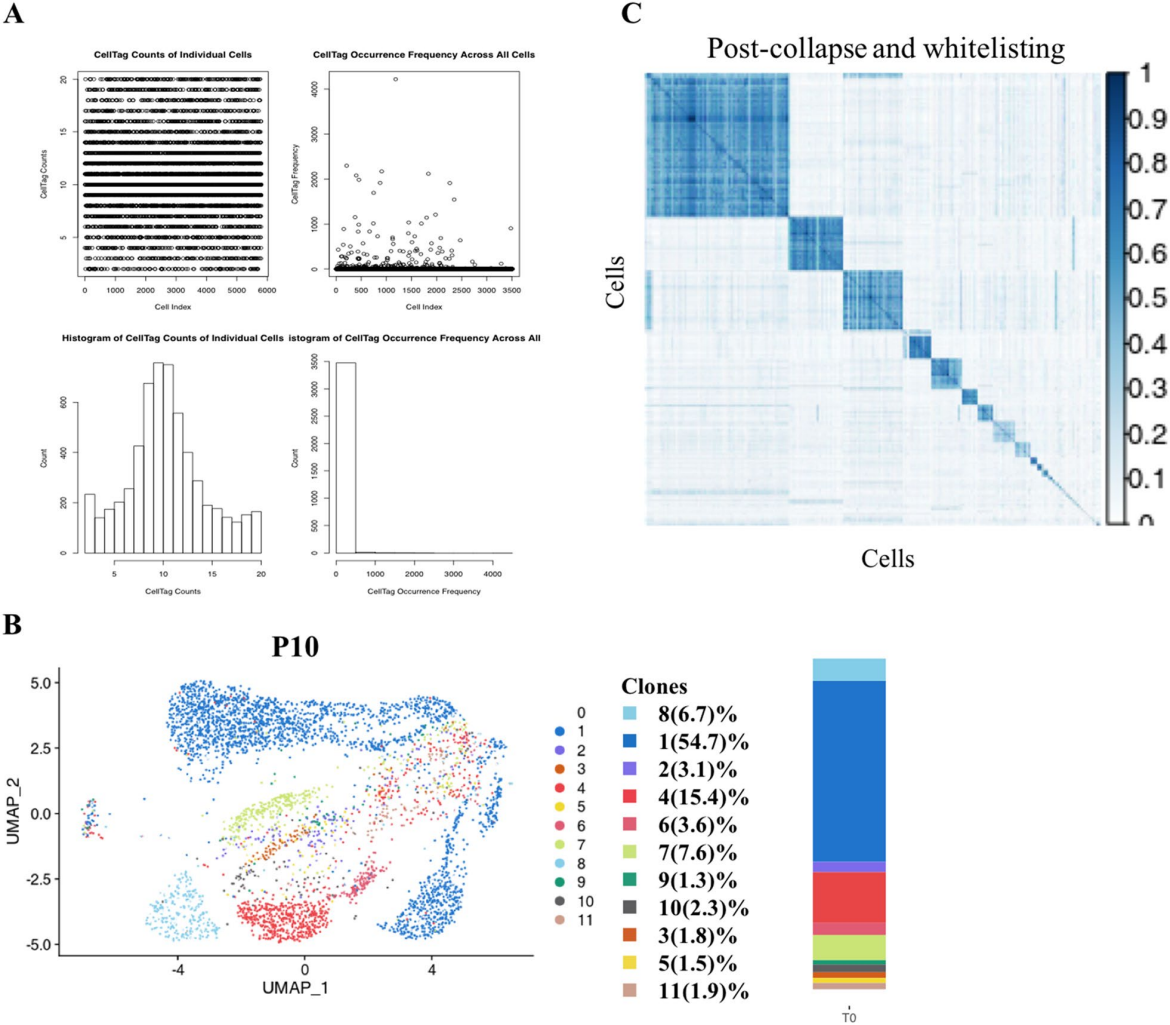
(**A**) Generation of the CellTag whitelist. Following single cell RNA sequencing, CellTags were first extracted from the raw sequencing files and the number of CellTags that entered each cell were calculated. Cells were not uniquely labeled with high confidence if there was only one CellTag per cell. Hence, we let each cell be tagged with 2–20 CellTags to increase the specificity of each cell. Through detection, we found that most of the labeled cells expressed 5–15 CellTags. (**B**) Visual clone distribution of the P10 CellTag data: projection of CellTag clones onto the UMAP plot. The histogram shows the proportion of cells from different clones. (**C**) CellTag signatures were extracted for each cell, and the overlap of CellTag signatures between cells were assessed using the Jaccard similarity analysis. Hierarchical clustering of cells based on each cell’s Jaccard similarity index with other cells to define ‘block’ of cells that originated from a single clone

To visualize cloning-related cell distribution, we determined the distribution of clones by using CellTags combined with UMAP mapping of P10 samples. We first read and filtered CellTags which were contained in the whitelist and were ≥ 2 and ≤ 20 in number in a single cell (Fig. 3A). After that, we projected the CellTags on the P10 UMAP map (Fig. 3B, C). From the figure, we found that the number of cells contained in different clones was very different, and that the number in the blue clone (clone 1) was significantly higher than that in other clones, which indicated that the growth rate of different cells was different. It is worth noting that blue clone (clone 1) grown from one cell was scattered in multiple subgroups, while the clones of other colors were distributed in a single subgroup, which indicates that the MSCs spontaneously differentiate into different types during the passage in vitro. A single cell has the potential of one-way and multi-way differentiation. This result further confirmed that hADSCs can expand the population of xenogenic MSCs in vitro.

### Candidate subgroup characteristics of the P15 sample

In order to study how cell heterogeneity affects differentiation, we aimed to induce hADSCs to enter the endoderm and track the differentiation. We added the growth factors activinA and Wnt3a to the P10 cells to stimulate them, and after 5 days, single cell sequencing of the stimulated cells was carried out. Through CellTag analysis, we identified the differentiation path of hADSC clones and made the differentiated cells correspond to those before differentiation. P10 cells started to differentiate in all directions, except the early undifferentiated state; they continued to differentiate into the mesoderm with the addition of endoderm growth factors.

**Fig. 4:**
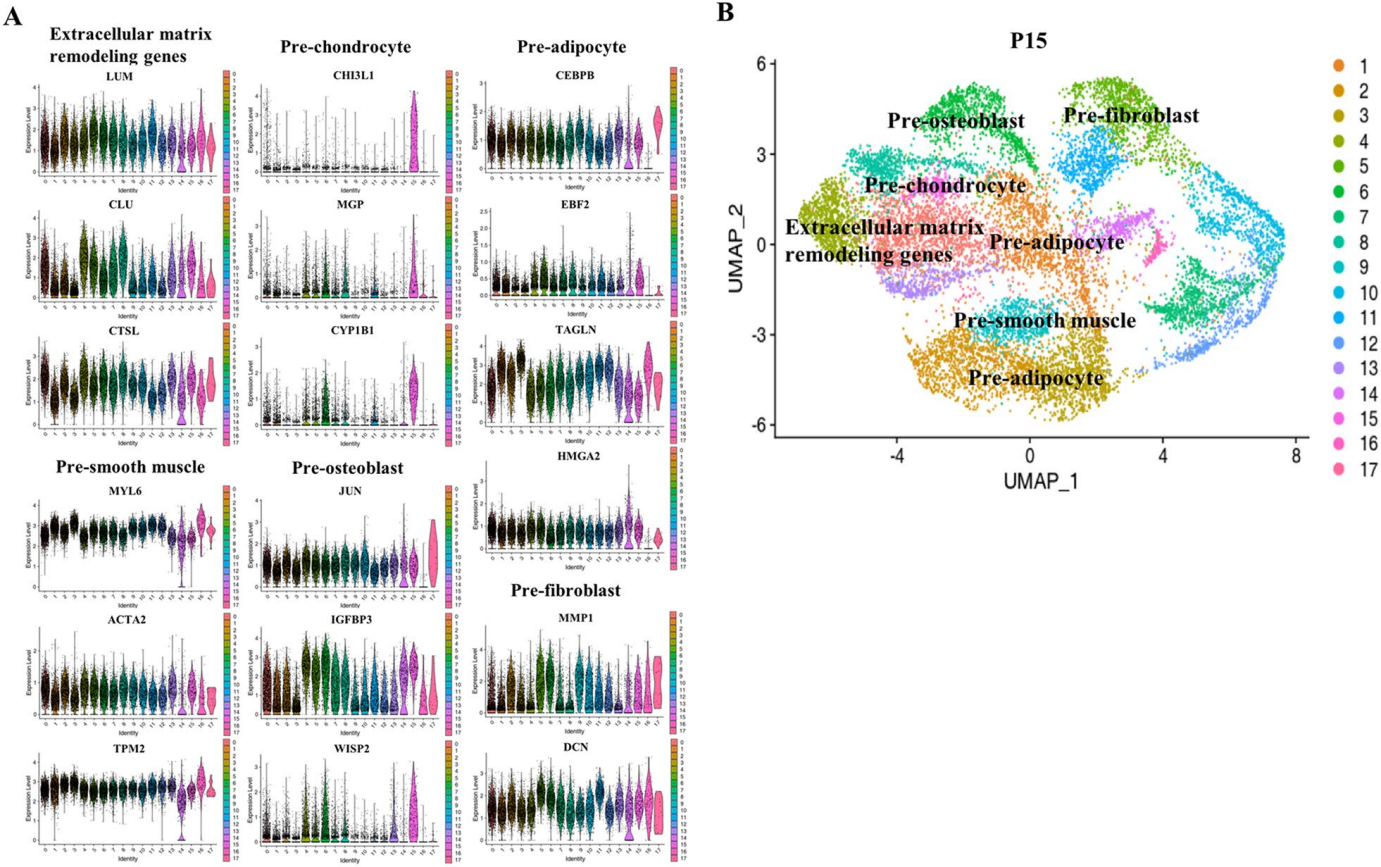
Differential gene expression and clustering map of induced hADSCs. (**A**) Violin plots showing the expression of differential signature genes in each subpopulation. (**B**) UMAP plot analysis of differentiated hADSCs

We analyzed the differential genes of P15 cells. On the basis of differential gene expression, we identified that cluster CC5 mainly expressed the fibroblast-related genes *MMP1* [30] and *DCN* [31]; cluster CC6 mainly expressed genes related to osteogenic differentiation regulation, such as *JUN* [34], Insulin Like Growth Factor Binding Protein 3 (*IGFBP3*) [36], and Cellular Communication Network Factor 5 (*WISP2*) [37]; cluster CC15 mainly expressed chondrocyte-related genes *CHI3L1* [29], Matrix Gla Protein (*MGP*) [38], and *CYP1B1* [28]; cluster CC0, CC4, CC8, and CC13 mainly expressed extracellular matrix-related genes; cluster CC9 expressed smooth muscle-related genes *MYL6* [26], *ACTA2* [25], and *TPM2* [27]; and clusters CC1, CC2, CC3, and CC14 mainly expressed adipose-related genes, such as CCAAT Enhancer Binding Protein Beta (*CEBPB*) [39], EBF Transcription Factor 2 (*EBF2*) [40], *TAGLN* [32], and *HMGA2* (Fig. 4A, B) [33]. Contrary to our expectations, we found that the hADSCs (P10) did not differentiate into the endoderm but matured further into the mesoderm under the stimulation of endodermal induction, and the expression of the more mature stage marker gene was detected in hADSCs (P15).

We found that most of the differentiated cells belonged to the same clone. This explained great multidirectional differentiation ability in different clusters among one clone. In addition, we found that clusters CC10 and CC12 only belonged to one clone. This proved that some cells can specifically differentiate into osteoblasts. In order to clarify the differentiation of these and other cells, we selected these cells and tested the differential expression of genes to determine which genes were significantly enriched and which may be the key regulatory genes affecting complete induction.

### Cloning-related cell distribution of the P15 sample

**Fig. 5:**
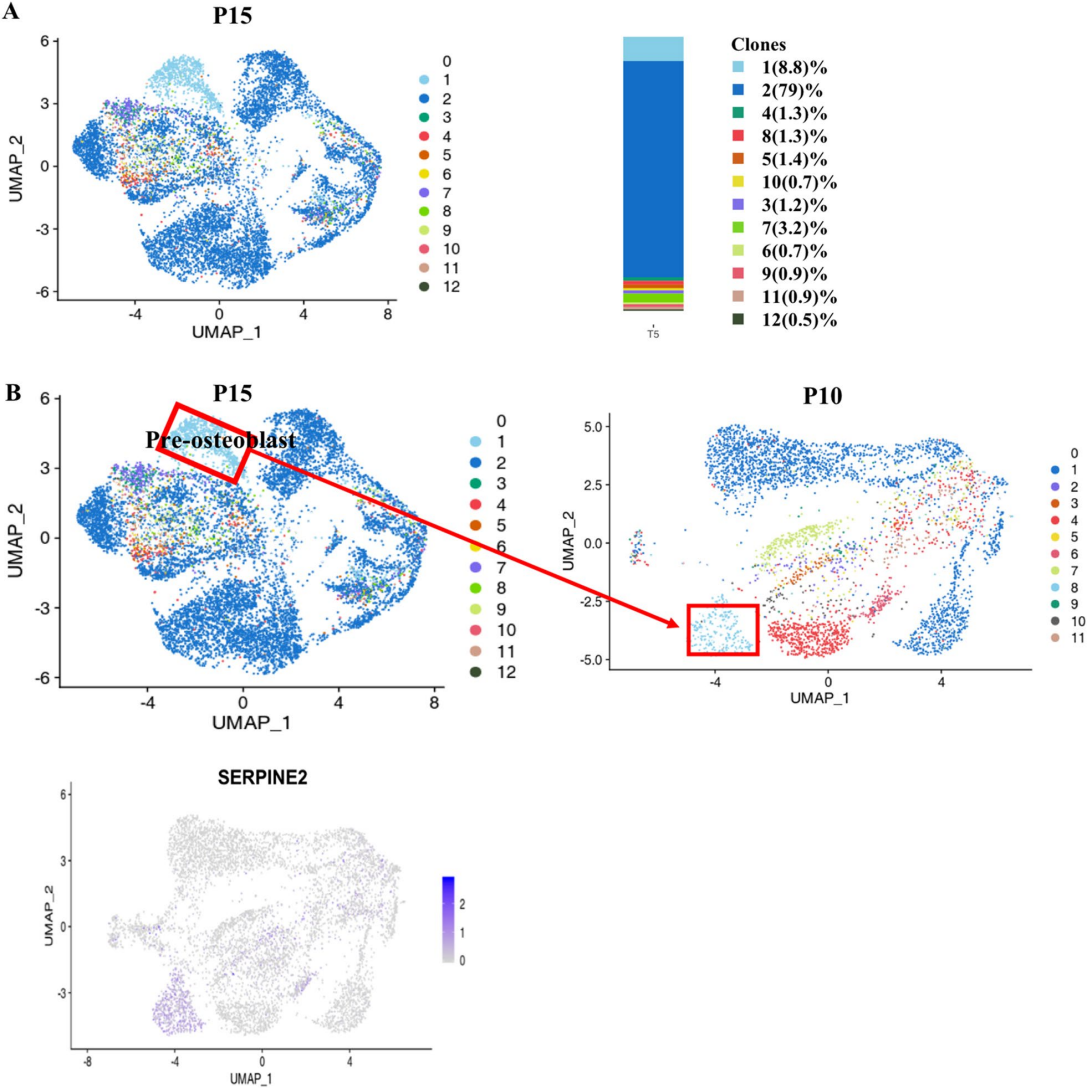
(**A**) Visual clone distribution of P15 CellTag data: projection of CellTag clones onto the UMAP plot. The histogram shows the proportion of cells from different clones. (**B**) CellTag was used to reconstruct the trajectory before and after lineage differentiation, and a cluster of cells related to osteogenic differentiation was found in the pre-induction cells

In order to visualize the cell distribution related to the P15 cloning and identify the difference before and after stimulation, we removed the CellTag of P15, projected it on the UMAP map, drew the clone distribution map (Fig. 5A), and compared the clone distribution of P10 and P15. As can be seen from the figure, the proportion of blue clone (Clone 2) was higher after growth factor stimulation, indicating that different clones had different proliferative abilities. Compared with other clones, the distribution of blue clone (Clone 2) also had more subgroups, indicating that different clones had different proliferative and differentiation abilities.

### Lineage reconstruction and identification of differentiation trajectories

In order to reconstruct the lineage of the cell clones before and after the stimulation, we projected CellTag on the UMAP map (Fig. 5B). From the figure, we found that there was a subgroup belonging to a single clone, which indicated that the cells of this subgroup were differentiated from one cell. Therefore, we aimed to identify this group of cells and used CellTag to track their differentiation. We found that this group of specific cells is related to osteogenic differentiation and proved that some cells can specifically differentiate into osteoblasts. Through CellTag, we picked out the barcodes of these cells that belonged to the pre-induction cells and positioned them in the pre-induction cell subgroups. We found that they were concentrated in one subgroup. In order to clarify the difference between these and other cells, we analyzed the gene differential expression between these cells and the remaining cells to determine which genes in these cells were significantly enriched and could be considered key regulators affecting osteogenesis and differentiation.

### Early stages delineate successful induction

To investigate the molecular characteristics underpinning the distinct inducing paths, we compared gene expression of cells differentiated into osteoblasts and that of non-osteogenic cells. Between these two tracks, remarkable changes of gene expression were obvious, including changes in *WNT*, ADP Ribosylation Factor 6 (*ARF6*)*,* and TAp63 Promoter Of Tumor Protein P63 (*TAP63*) signal pathways. The non-osteogenic differentiation trajectory enriched the expression of the Hyaluronan And Proteoglycan Link Protein 1 (*HAPLN1*) [41] and Collagen Type XI Alpha 1 Chain (*COL11A1*) [42] genes, which was consistent with the reactivation of chondrogenic differentiation. The osteogenic differentiation trajectory was found to be regulated by Prostaglandin-Endoperoxide Synthase 1 (*PTGS1*) [43], Cell Migration Inducing Hyaluronidase 1 (*CEMIP*) [44], and Dehydrogenase/Reductase 3 (*DHRS3*) [45], which supports our observations that these results were established from early stages. In addition to some genes with known functions, we found that there were significant differences in many unreported genes, such as *SERPINE2* [46], *SFRP1*, *KRT7*, *PI16*, and *CPE*, which may be potential targets to optimize the efficiency of osteogenic induction [47].

### SERPINE2 promotes the osteoblastic differentiation of hADSCs

**Fig. 6:**
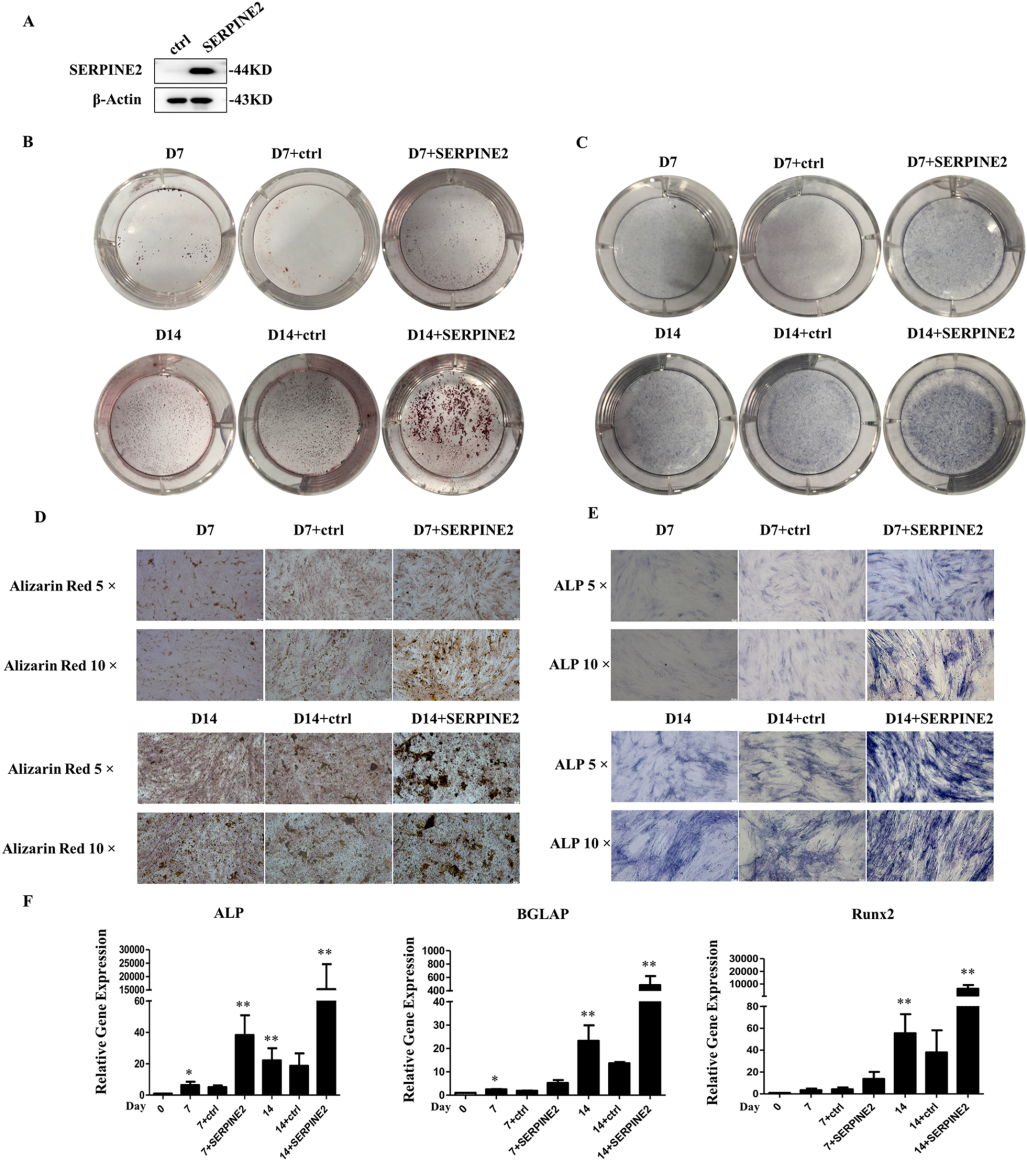
(**A**) Western blot analysis shows that the expression of SERPINE2 was upregulated after its overexpression, full-length blots are presented in Additional file 1: Fig. S1 (**B**, **C**, **D**, **E**) Activation of SERPINE2 promotes osteogenic differentiation of hADSCs, as observed under the microscope. Alizarin Red staining shows that the cell mineralization ability was enhanced after activating SERPINE2 compared with the control group. ALP staining shows an increase in ALP content after overexpression of SERPINE2 compared to the control group. (**F**) qRT-PCR was used to detect the expression levels of the osteogenesis-associated genes *ALP*, *BGLAP*, and *Runx2* during the osteoblast differentiation of hADSCs

In order to clarify the effect of *SERPINE2* on osteoblast differentiation, hADSCs were infected with lentivirus, and SERPINE2 was overexpressed (Fig. 6A). After 7 and 14 days of induction, Alizarin Red S and alkaline phosphatase (ALP) were used as differentiation markers of osteoblasts. Alizarin Red S staining indicates mineralized nodule formation in hADSCs. In the present study, staining with Alizarin Red S and ALP was stronger in cells induced for 14 days to differentiate into osteoblasts than that in the group induced for 7 days. Furthermore, these two markers were upregulated in *SERPINE2*-infected cells on the 7th and 14th day (Fig. 6B–E). In addition, RT‐PCR showed that the expression levels of RUNX Family Transcription Factor 2 (*Runx2*), Bone Gamma-Carboxyglutamate Protein (*BGLAP*), and Alkaline phosphatase (*ALP*) were significantly increased in the *SERPINE2*-infected group (Fig. 6F). All these results indicated that *SERPINE2* accelerated the osteoblast differentiation of hADSCs.

## Discussion

**Fig. 7:**
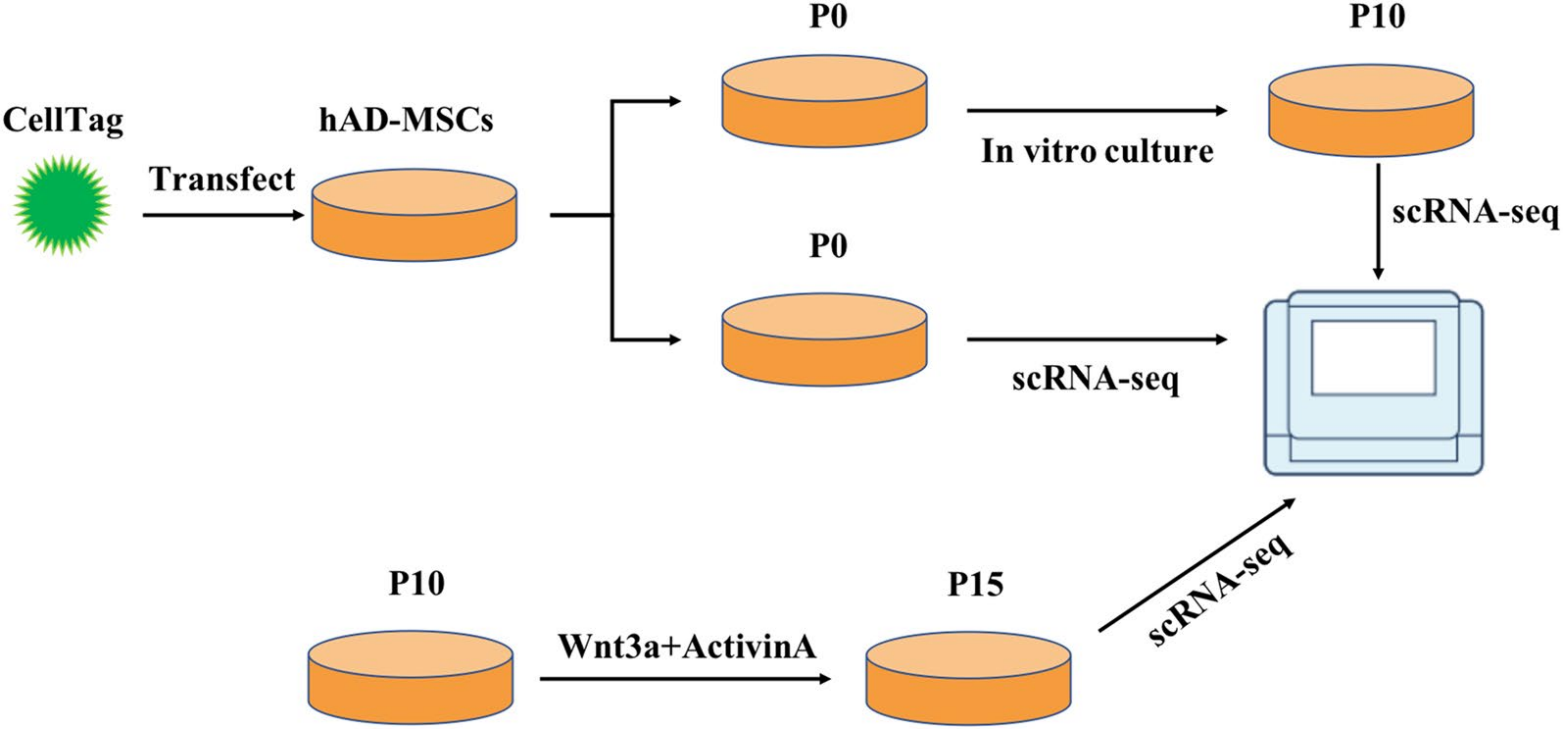
Experimental design to trace the differentiation trajectory of hADSCs

In this study, the heterogeneity and differentiation potential of MSCs were explored by combining scRNA-seq and CellTagging (Fig. 7). Firstly, according to the results of scRNA-seq, the distribution of subgroups is relatively concentrated in the early stage and discrete with a multi-directional differentiation pattern with the passages in vitro, which showed that with in vitro passaging, the heterogeneity of MSC increases, which is related to the spontaneous differentiation of cells during passaging [23]. From the detected CellTags, it can be seen that some clones with the same combination of CellTags have appeared in different subpopulations, it is further proved that early hADSCs undergo spontaneous differentiation with in vitro amplification. These results suggest that early MSC have the potential of multi-differentiation, and the in vitro culture conditions are not well enough to maintain the stemness of MSC. The amplification system needs further improvement.

The feasibility of CellTagging technology in tracking the differentiation of MSCs was also proven. Due to the limitation of current CellTagging technology, the detection of CellTags should be amplified to a sufficient number. So the cells have been subcultured in vitro to the tenth generation when we tagged them. Using CellTagging technology, we found that the stimulated clones were mainly divided into two parts. One part of the clones was dominant with strong proliferation and differentiation potential, and the other was specifically related to osteogenic differentiation from beginning. This osteogenic differentiation specific cluster might be generated during the early passages.

Analyzing the heterogeneity of MSCs is beneficial to clinical treatment. Cell therapy is a new technology for new drug research and development. Developing MSCs with real clinical effects is the goal of scientists. However, the unstable therapeutic effect of MSCs, lack of clear mechanism research, and lack of understanding of the fate of cellular drugs in the body hinder the utilization of its medicinal properties [48]. Studying the heterogeneity of MSCs can help us to better understand their differences and improve their therapeutic effects, which is of great help to reduce the uncertainty of MSCs as therapeutic drugs [49].

The adipogenic and osteogenic differentiation abilities are the most common characteristics of mesenchymal stem cells [50–53]. We have focused on osteogenesis and adipogenesis of mesenchymal stem cells. Our CellTagging results show that a cluster of cells may be poised for osteoblast differentiation from the early stage. Therefore, we focused on osteogenic differentiation, and our findings revealed several potential candidates for osteogenic differentiation: The *SERPINE2* gene has a significant effect on osteogenic differentiation, which further improves the efficiency and fidelity of cell induction. Besides being valuable for the biological research of MSCs, this information is also of great significance for the standardization of MSCs and further development of strategies for treating various diseases using MSCs. SERPINE2, also known as protease nexin-1 (PN-1), is a member of the serine protease inhibitor superfamily, and is a secretory protein with anti-serine protease activity of antithrombin, urokinase, plasminogen, and other serine proteases [46, 54]. Previous studies have shown that the change of SERPINE2 expression could increase expression of bone morphogenetic protein 4 (*BMP4*), thus, activating the BMP signal. BMPs play an important role in the formation of bones [55]. Hence, activating SERPINE2 might enhance BMP-mediated bone formation.

The next challenge will be to reveal the molecular characteristics of these potential candidate cells to further improve the ability of inducing cells to achieve any desired cell identity with high efficiency and fidelity.

## Conclusions

Combined with CellTagging technology and single cell sequencing, our results show that MSC has the potential of multi-directional differentiation, and the heterogeneity in vitro will increase with cell passages, suggesting that the in vitro culture and amplification conditions of MSC need to be improved. We reported for the first time that SERPINE2 can promote osteogenic differentiation of MSCs.

## Supplementary Information

**Additional file 1**. **Figure S1** The figure shows the original uncropped western blot images and the red box shows the cropped images in the main text. (**A**) Figure shows the blank film with marker position. (**B**) Figure shows the original and transverse stretched strips of SERPINE2. (**C**) Figure shows the original strips of β-actin.

**Additional file 2.** The RNA primers applied for qRT-PCR.

## Data Availability

All the data are included in the manuscript.

## Abbreviations

MSCs: Mesenchymal stromal cells
ADSCs: Adipose tissue derived stem cells
SERPINE2: Serpin Family E member 2
scRNA-seq: Single cell transcriptome sequencing
BMSCs: MSCs derived from bone marrow
BM: Basic medium
OM: Osteogenic medium
GEO: Gene Expression Omnibus
SFRP1: Secreted Frizzled Related Protein 1
KRT7: Keratin 7
PI16: Peptidase Inhibitor 16
CPE: Carboxypeptidase E
BMP4: Bone morphogenetic protein 4
ACTA2: Actin Alpha 2
MYL6: Myosin Light Chain 6
TPM2: Tropomyosin 2
CYP1B1: Cytochrome P450 Family 1 Subfamily B Member 1
CHI3L1: Chitinase 3 Like 1
TRPS1: Transcriptional Repressor GATA Binding 1
MMP1: Matrix Metallopeptidase 1
DCN: Decorin
TAGLN: Transgelin
HMGA2: High Mobility Group AT-Hook 2
ATF4: Activating Transcription Factor 4
JUN: Jun Proto-Oncogene, AP-1 Transcription Factor Subunit
UMAP: Uniform Manifold Approximation and Projection
IGFBP3: Insulin Like Growth Factor Binding Protein 3
WISP2: Cellular Communication Network Factor 5
MGP: Matrix Gla Protein
CEBPB: CCAAT Enhancer Binding Protein Beta
EBF2: EBF Transcription Factor 2
Runx2: RUNX Family Transcription Factor 2
BGLAP: Bone Gamma-Carboxyglutamate Protein
ALP: Alkaline phosphatase
BMP4: Bone morphogenetic protein 4
PN-1: Protease nexin-1
