# Supplementary figures and images for: Combining single-cell transcriptomics and CellTagging to identify differentiation trajectories of human adipose-derived mesenchymal stem cells
Source: Stem Cell Res Ther. 2023 Feb 1;14:14. doi: 10.1186/s13287-023-03237-3 (PMC9890798; doi:10.1186/s13287-023-03237-3)

A

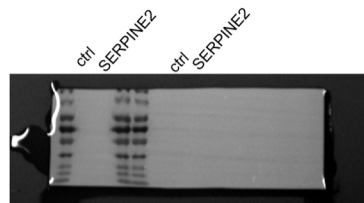

B

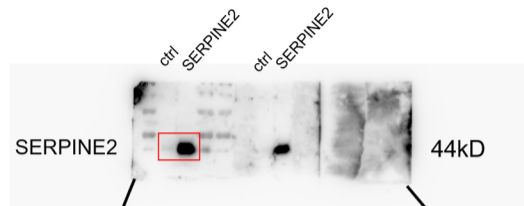

C

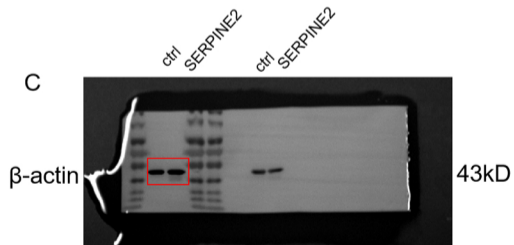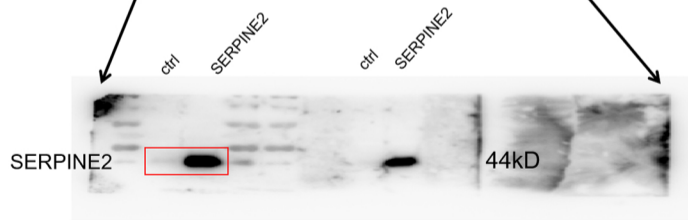

Supplement: Supplementary file 1 — Additional file 1. Figure S1 The figure shows the original uncropped western blot images and the red box shows the cropped images in the main text. (A) Figure shows the blank film with marker position. (B) Figure shows the original and transverse stretched strips of SERPINE2. (C) Figure shows the original strips of β-actin. [file 13287_2023_3237_MOESM1_ESM.pdf]
