# Supplementary material for: Combining single-cell transcriptomics and CellTagging to identify differentiation trajectories of human adipose-derived mesenchymal stem cells
Source: Stem Cell Res Ther. 2023 Feb 1;14:14. doi: 10.1186/s13287-023-03237-3 (PMC9890798; doi:10.1186/s13287-023-03237-3)
Supplement: Supplementary file 2 — Additional file 2. The RNA primers applied for qRT-PCR. [file 13287_2023_3237_MOESM2_ESM.docx]

Supplementary Table 1

| Gene name | Sequence |
| --- | --- |
| Human Runx2 | Forward primer 5’—3’GCAAGGTTCAACGATCTGAG |
|  | Reverse primer 5’—3’GGAGGATTTGTGAAGACGGT |
| Human ALP | Forward primer 5’—3’CCTAGTTATTGCCCTTTGGCC |
|  | Reverse primer 5’—3’TGCCTGCCCAAGAGAGAAA |
| Human BGLAP | Forward primer 5’—3’GGCGCTACCTGTATCAATGG |
|  | Reverse primer 5’—3’GTGGTCAGCCAACTCGTCA |
| Human GAPDH | Forward primer 5’—3’GAAGGTGAAGGTCGGAGTC |
|  | Reverse primer 5’—3’ GAAGATGGTGATGGGATTTC |
